# Supplementary material for: Advancements in Hyperspectral Imaging and Computer-Aided Diagnostic Methods for the Enhanced Detection and Diagnosis of Head and Neck Cancer
Source: Biomedicines. 2024 Oct 11;12(10):2315. doi: 10.3390/biomedicines12102315 (PMC11504349; doi:10.3390/biomedicines12102315)
Supplement: Supplementary file 1 [file biomedicines-12-02315-s001.zip › biomedicines-3196742-supplementary.pdf]

S1.Literature search

Authors (A.M, G.G) independently searched for articles on the web, using Google Scholar, for this study. They selected literature from 2015 to 2024. Duplicate articles were excluded. They reviewed titles and abstracts to ensure articles were relevant. Additionally, full-text reviews were conducted to determine if articles met inclusion criteria.

S1.1. Inclusion criteria

This review aims to concentrate on studies meeting specific inclusion criteria:

1. Studies should present clear numerical results like dataset, sensitivity, accuracy and specificity.
2. They should focus on hyperspectral imaging for Head and Neck cancer detection.
3. They must be published within the last nine years.
4. The publication journal should have an H-index exceeding 70 and belong to the first quartile (Q1) or second quartile(Q2).
5. They should have a prospective or retrospective design.
6. The studies should be written in English.

S1.2. Exclusion criteria

This review will exclude studies that meet the following criteria:

1. Studies lacking sufficient data.
2. Narrative, systematic review, and meta-analysis studies.
3. Comments, proceedings, or study protocols.
4. Conference papers.

S1.3. Data extraction, primary outcomes and additional analyses

Authors (A.M and G.G) conducted data extraction and cross-checking. Email served as the primary mode of communication for data inquiries and validation. Each study was synthesized through a diagnostic test accuracy (DTA) and systematic review process. Meta-analyses primarily focused on accuracy, sensitivity, and specificity of diagnostic performance using endoscopic imaging in each study.

Additionally, the geographical origin of data was documented for subgroup analysis purposes. The subgroup table included information on CAD methods, parts of the body, Band, Vivo and Year of publication.

S1.4. Study Inclusion

A total of 1030 results were identified through a Google Scholar search. After considering the publication years, 101 articles were excluded as the review aimed to focus solely on articles published between 2015 and 2024. Additionally, articles lacking full-text access, totaling around 397, were observed and also considered for exclusion. This left a total of 532 articles for review. Among these, 525 records were excluded due to reasons such as

incomplete data, narrative reviews, meta-analyses, comments, and conference papers, which were part of the exclusion criteria. Ultimately, only 7 studies met the inclusion criteria and were included in this review. figure below shows a flow chart for selection processes.

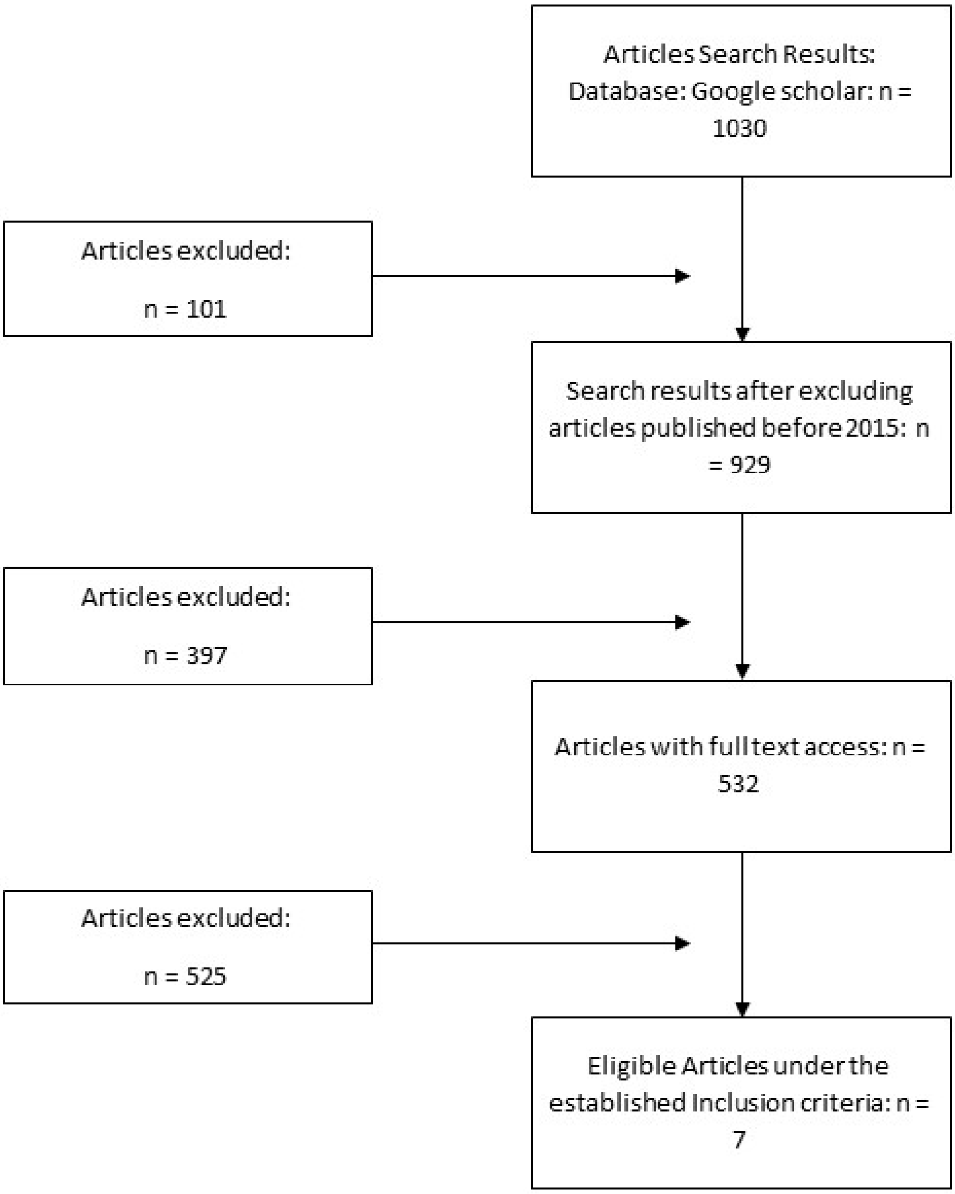

Fig S1. Flowchart based on diagnostic cohort study of HNC diagnosis

S2. QUADAS-2

This section provides a summary of the QUADAS-2 assessment results for the eight studies included in this review. It outlines the concerns regarding applicability and the degree of bias risk observed across the studies concerning flow and timing, patient selection, reference standard, and index test. Each study underwent evaluation for bias risk in flow and timing, patient selection, reference standard, and index test, as well as for applicability concerns related to patient selection, reference standard, and index test.

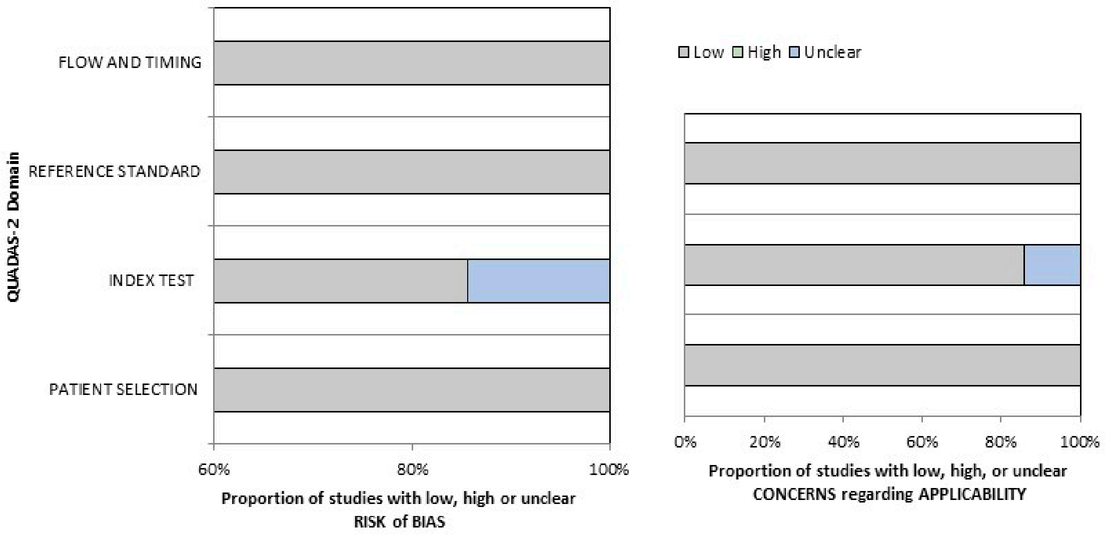

Fig S2 . Quadas-2 results

S3. Forest plot

Forest plots of accuracy, sensitivity and specificity for different categories including cancer types, band, vivo, year of publication and CAD methods are shown below. the analyses were performed at 95% confidence level.

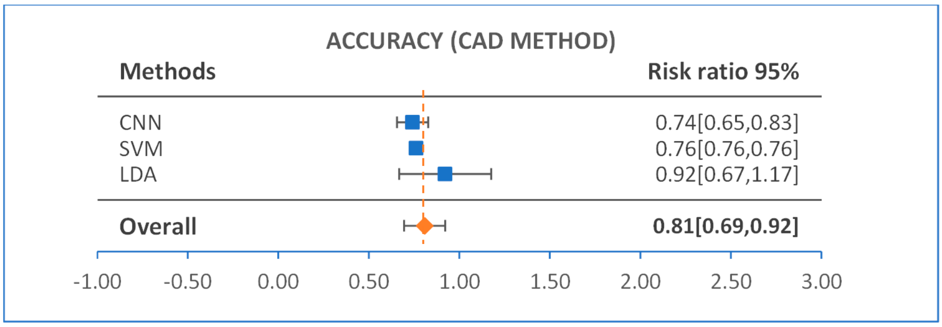

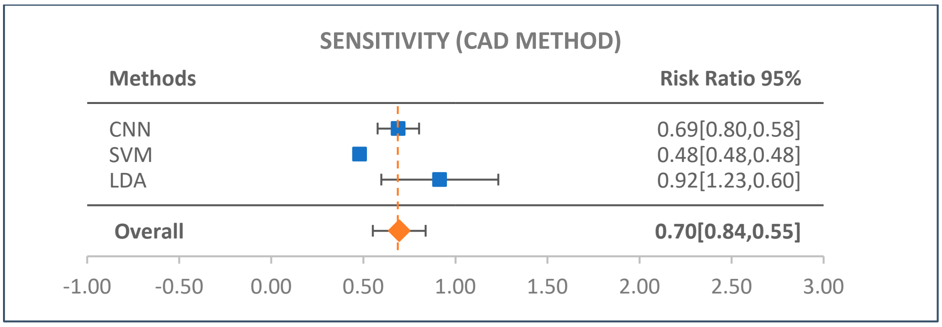

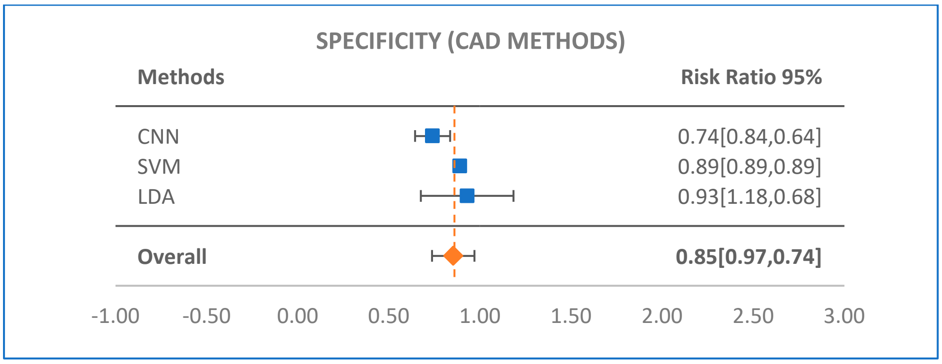

Forest plots for CAD Methods

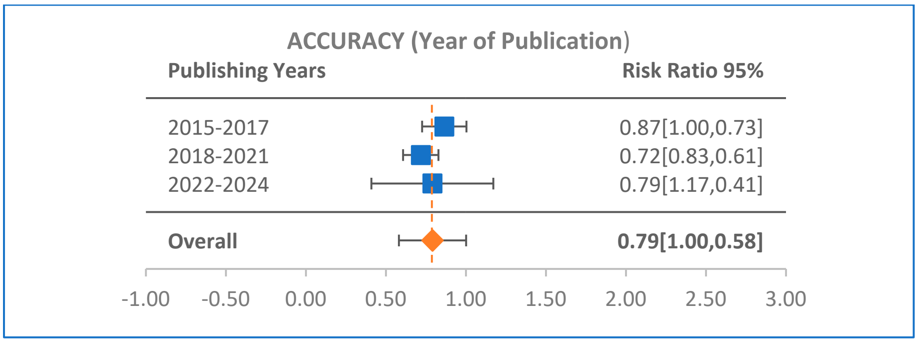

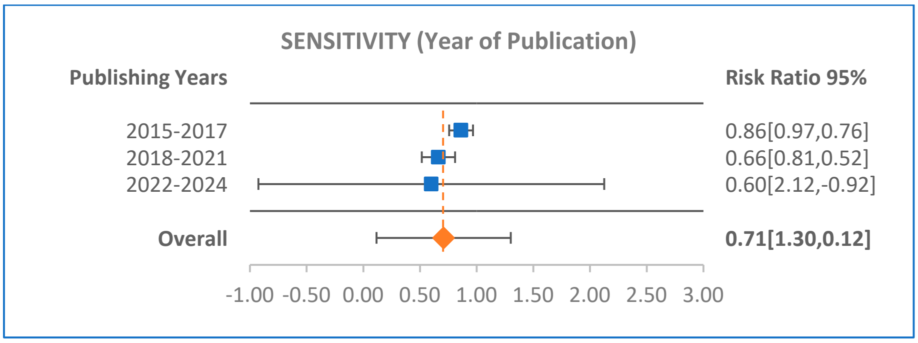

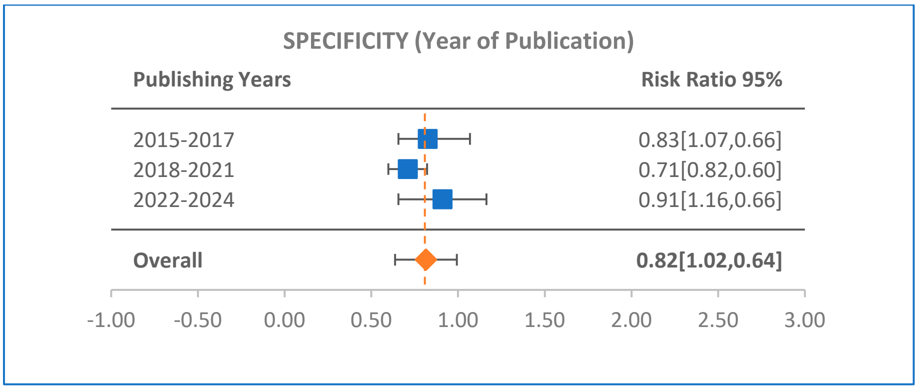

Forest plots for publication Year

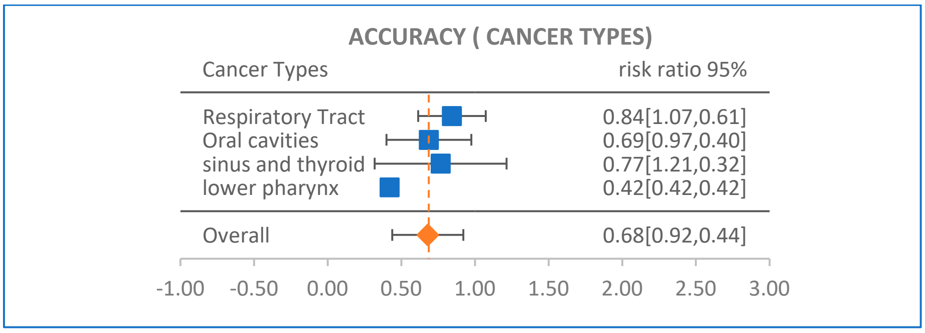

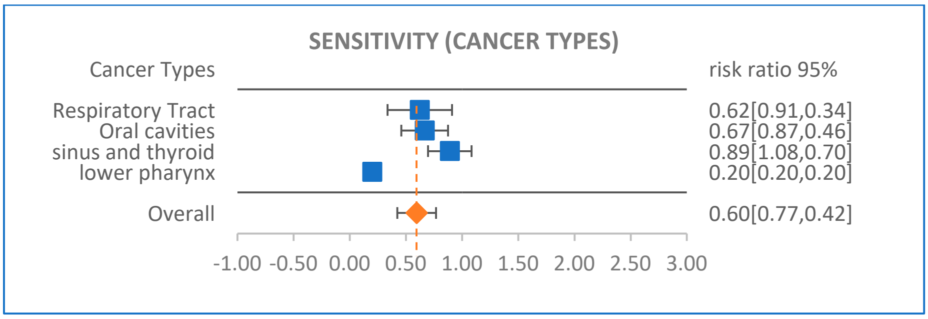

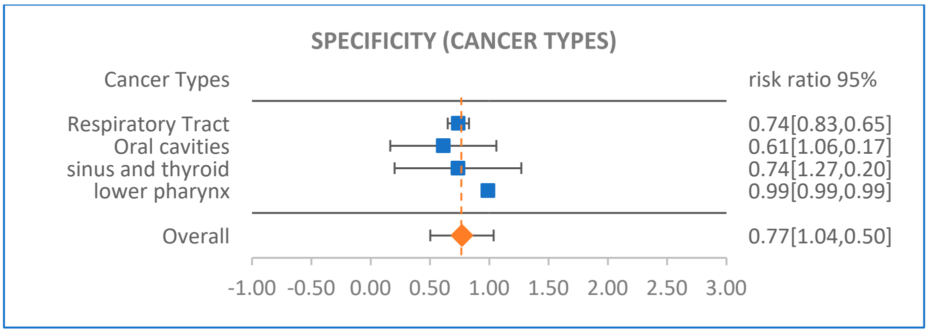

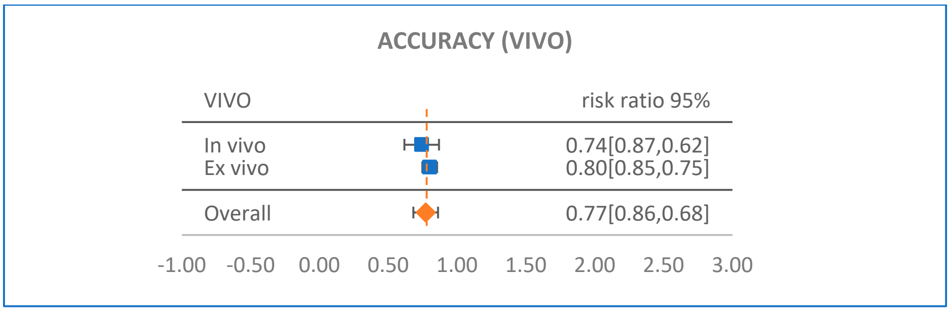

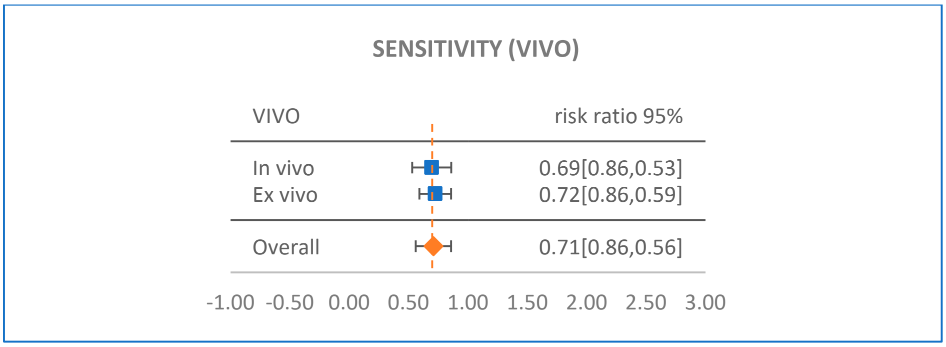

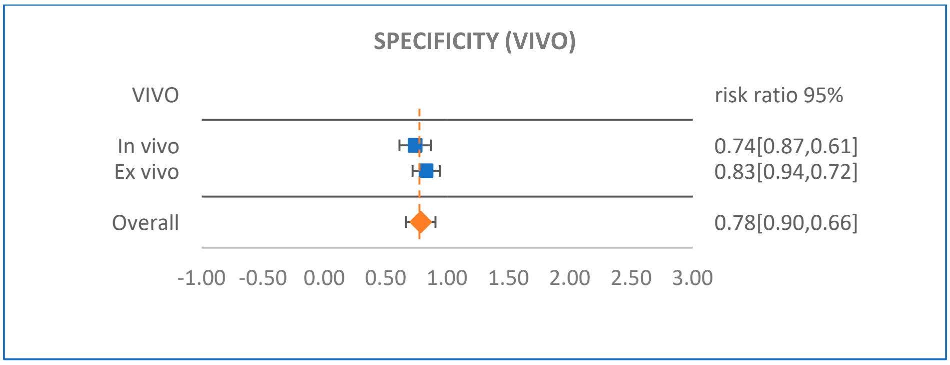

Figure S3. Forest plots

S4. Deek's plot

This section presents deek's funnel plots for vivo, band, CAD methods. Year of publication and cancer types.

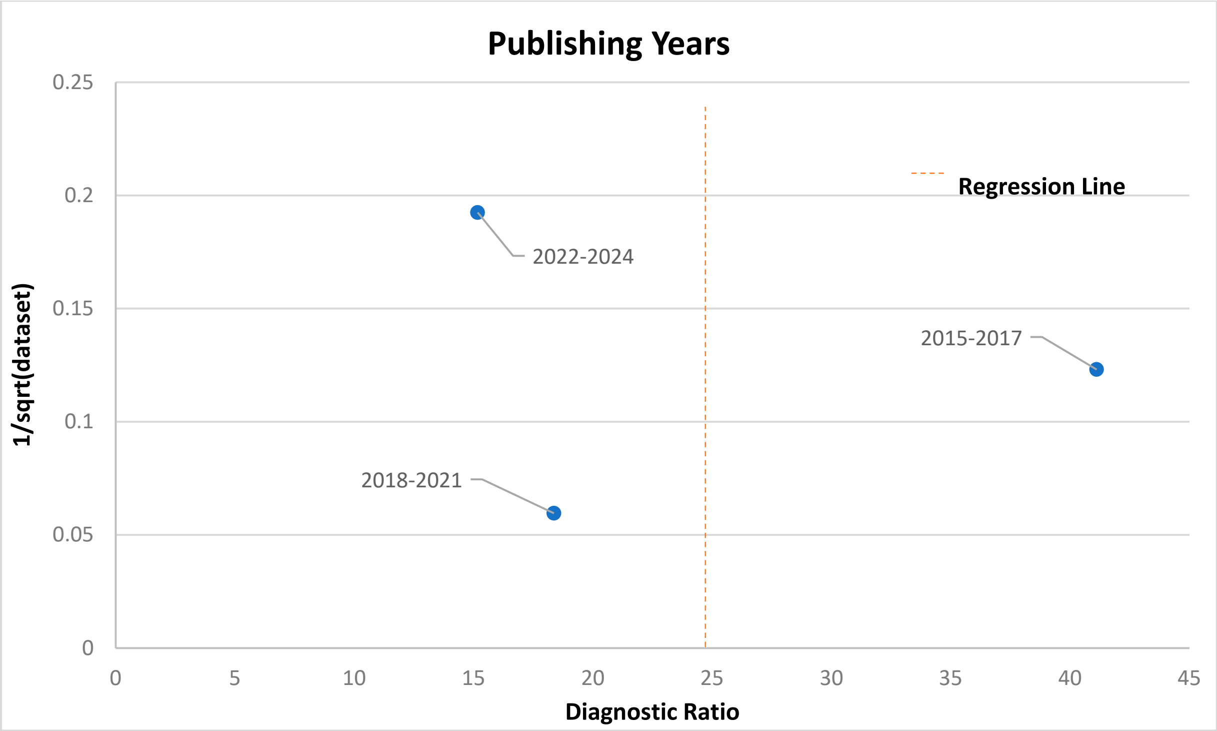
Publishing Years
Regression Line
2022-2024
2015-2017
2018-2021
1/sqrt(dataset)
Diagnostic Ratio
0.25
0.2
0.15
0.1
0.05
0
0 5 10 15 20 25 30 35 40 45

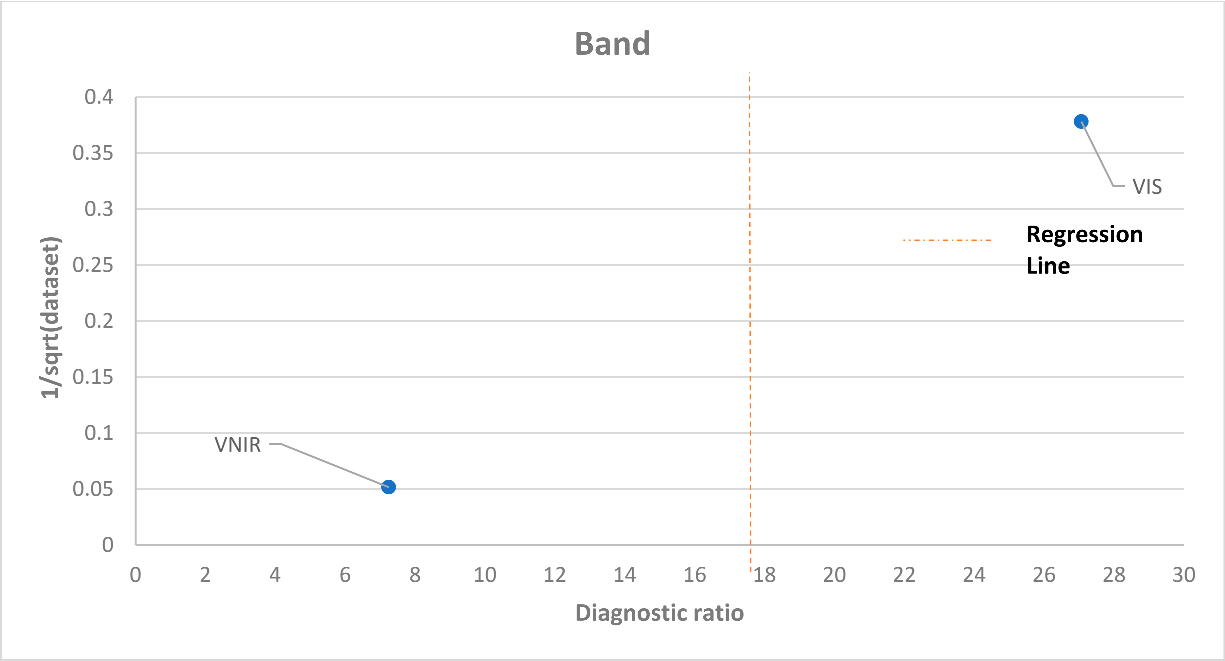
Band
VIS
Regression Line
VNIR
1/sqrt(dataset)
Diagnostic ratio
0.4
0.35
0.3
0.25
0.2
0.15
0.1
0.05
0
0 2 4 6 8 10 12 14 16 18 20 22 24 26 28 30

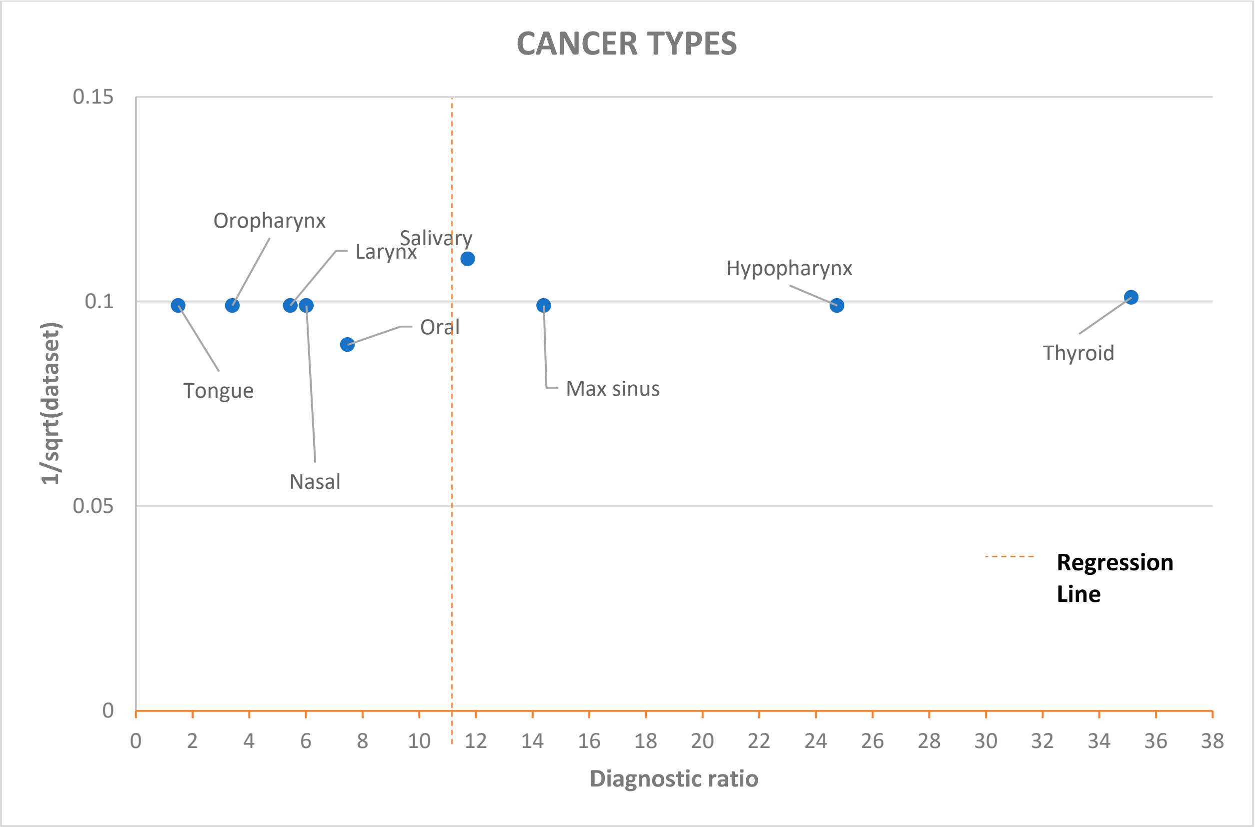

Fig S4. Deek's Funnel Plot

S5. Computations for forest plot

ACCURACY (2022-2024)

Column1	
Mean	79
Standard Error	3
Median	79
Mode	#N/A
Standard Deviation	4.242641
Sample Variance	18
Kurtosis	#DIV/0!
Skewness	#DIV/0!
Range	6
Minimum	76
Maximum	82
Sum	158
Count	2
Confidence Level (95.0%)	38.11861
**UPPER CI**	**117.1186**
**LOWER CI**	**40.88139**

SENSITIVITY (2022-2024)

Column1	
Mean	60
Standard Error	12
Median	60
Mode	#N/A
Standard Deviation	16.97056
Sample Variance	288
Kurtosis	#DIV/0!
Skewness	#DIV/0!
Range	24
Minimum	48
Maximum	72
Sum	120
Count	2
Confidence Level (95.0%)	152.4745
**UPPER CI**	**212.4745**
**LOWER CI**	**-92.4745**

SPECIFICITY (2022-2024)

Column1	
Mean	91
Standard Error	2
Median	91
Mode	#N/A
Standard Deviation	2.828427
Sample Variance	8
Kurtosis	#DIV/0!
Skewness	#DIV/0!
Range	4
Minimum	89
Maximum	93
Sum	182
Count	2
Confidence Level(95.0%)	25.41241
**UPPER CI**	**116.4124**
**LOWER CI**	**65.58759**

ACCURACY (2018-2021)

Column1	
Mean	71.8
Standard Error	4.878069

SENSITIVITY (2018-2021)

Column1	
Mean	66.3
Standard Error	6.479112

SPECIFICITY (2018-2021)

Column1	
Mean	71
Standard Error	4.944132

Median	78.5
Mode	79
Standard Deviation	15.42581
Sample Variance	237.9556
Kurtosis	0.128209
Skewness	-0.59484
Range	53
Minimum	42
Maximum	95
Sum	718
Count	10
Confidence Level(95.0%)	11.03496
**UPPER CI**	**82.83496**
**LOWER CI**	**60.76504**

Median	70
Mode	69
Standard Deviation	20.48875
Sample Variance	419.7889
Kurtosis	2.243816
Skewness	-1.26994
Range	73
Minimum	20
Maximum	93
Sum	663
Count	10
Confidence Level(95.0%)	14.65677
**UPPER CI**	**80.95677**
**LOWER CI**	**51.64323**

Median	73.5
Mode	#N/A
Standard Deviation	15.63472
Sample Variance	244.4444
Kurtosis	-0.30154
Skewness	0.030308
Range	50
Minimum	49
Maximum	99
Sum	710
Count	10
Confidence Level(95.0%)	11.1844
**UPPER CI**	**82.1844**
**LOWER CI**	**59.8156**

ACCURACY (2015-2017)

Column1	
Mean	86.5
Standard Error	4.349329
Median	89
Mode	#N/A
Standard Deviation	8.698659
Sample Variance	75.66667
Kurtosis	2.646199
Skewness	-1.50411
Range	20

SENSITIVITY (2015-2017)

Column1	
Mean	86.25
Standard Error	3.300884
Median	86
Mode	#N/A
Standard Deviation	6.601767
Sample Variance	43.58333
Kurtosis	-2.02496
Skewness	0.164218
Range	15

SPECIFICITY (2015-2017)

Column1	
Mean	86.25
Standard Error	6.472699
Median	91.5
Mode	#N/A
Standard Deviation	12.9454
Sample Variance	167.5833
Kurtosis	3.684124
Skewness	-1.89485
Range	28

Minimum	74
Maximum	94
Sum	346
Count	4
Confidence Level(95.0%)	13.84151
**UPPER CI**	**100.3415**
**LOWER CI**	**72.65849**

Minimum	79
Maximum	94
Sum	345
Count	4
Confidence Level(95.0%)	10.50489
**UPPER CI**	**96.75489**
**LOWER CI**	**75.74511**

Minimum	67
Maximum	95
Sum	345
Count	4
Confidence Level(95.0%)	20.59902
**UPPER CI**	**106.849**
**LOWER CI**	**65.65098**

ACCURACY (IN VIVO)

Column1	
Mean	74.2
Standard Error	5.543364
Median	79
Mode	79
Standard Deviation	17.52966
Sample Variance	307.2889
Kurtosis	-0.64942
Skewness	-0.51829
Range	53
Minimum	42
Maximum	95
Sum	742
Count	10
Confidence Level(95.0%)	12.53996
**UPPER CI**	**86.73996**

SENSITIVITY (IN VIVO)

Column1	
Mean	69.4
Standard Error	7.260548
Median	70
Mode	69
Standard Deviation	22.95987
Sample Variance	527.1556
Kurtosis	1.166608
Skewness	-1.0657
Range	74
Minimum	20
Maximum	94
Sum	694
Count	10
Confidence Level(95.0%)	16.4245
**UPPER CI**	**85.8245**

SPECIFICITY (IN VIVO)

Column1	
Mean	74
Standard Error	5.731007
Median	75.5
Mode	#N/A
Standard Deviation	18.12304
Sample Variance	328.4444
Kurtosis	-1.32684
Skewness	-0.13006
Range	50
Minimum	49
Maximum	99
Sum	740
Count	10
Confidence Level(95.0%)	12.96444
**UPPER CI**	**86.96444**

**LOWER CI**	**61.66004**

**LOWER CI**	**52.9755**

**LOWER CI**	**61.03556**

**ACCURACY (EX VIVO)**	
Column1	
Mean	80
Standard Error	2.065591
Median	80
Mode	82
Standard Deviation	5.059644
Sample Variance	25.6
Kurtosis	-0.16602
Skewness	0.555869
Range	14
Minimum	74
Maximum	88
Sum	480
Count	6
Confidence Level(95.0%)	5.309771
**UPPER CI**	**85.30977**
**LOWER CI**	**74.69023**

**SENSITIVITY (EX VIVO)**	
Column1	
Mean	72.33333
Standard Error	5.194014
Median	75.5
Mode	72
Standard Deviation	12.72268
Sample Variance	161.8667
Kurtosis	3.607699
Skewness	-1.8133
Range	35
Minimum	48
Maximum	83
Sum	434
Count	6
Confidence Level(95.0%)	13.35164
**UPPER CI**	**85.68497**
**LOWER CI**	**58.9817**

**SPECIFICITY (EX VIVO)**	
Column1	
Mean	82.83333
Standard Error	4.300517
Median	85.5
Mode	#N/A
Standard Deviation	10.53407
Sample Variance	110.9667
Kurtosis	-1.24019
Skewness	-0.66819
Range	26
Minimum	67
Maximum	93
Sum	497
Count	6
Confidence Level(95.0%)	11.05483
**UPPER CI**	**93.88816**
**LOWER CI**	**71.7785**

**ACCURACY (VNIR)**	
Column1	
Mean	75.64286

**SENSITIVITY (VNIR)**	
Column1	
Mean	69.5

**SPECIFICITY (VNIR)**	
Column1	
Mean	76.07143

Standard Error	4.032297
Median	78.5
Mode	79
Standard Deviation	15.08747
Sample Variance	227.6319
Kurtosis	0.342163
Skewness	-0.78664
Range	53
Minimum	42
Maximum	95
Sum	1059
Count	14
Confidence Level(95.0%)	8.711248
**UPPER CI**	**84.35411**
**LOWER CI**	**66.93161**

Standard Error	5.453852
Median	71.5
Mode	69
Standard Deviation	20.40645
Sample Variance	416.4231
Kurtosis	1.298041
Skewness	-1.07934
Range	74
Minimum	20
Maximum	94
Sum	973
Count	14
Confidence Level(95.0%)	11.78233
**UPPER CI**	**81.28233**
**LOWER CI**	**57.71767**

Standard Error	4.405871
Median	76
Mode	#N/A
Standard Deviation	16.48526
Sample Variance	271.7637
Kurtosis	-1.00467
Skewness	-0.36862
Range	50
Minimum	49
Maximum	99
Sum	1065
Count	14
Confidence Level(95.0%)	9.518305
**UPPER CI**	**85.58973**
**LOWER CI**	**66.55312**

ACCURACY (VIS)

Column1	
Mean	81.5
Standard Error	0.5
Median	81.5
Mode	#N/A
Standard Deviation	0.707107
Sample Variance	0.5
Kurtosis	#DIV/0!
Skewness	#DIV/0!

SENSITIVITY (VIS)

Column1	
Mean	77.5
Standard Error	5.5
Median	77.5
Mode	#N/A
Standard Deviation	7.778175
Sample Variance	60.5
Kurtosis	#DIV/0!
Skewness	#DIV/0!

ACCURACY (VIS)

Column1	
Mean	86
Standard Error	7
Median	86
Mode	#N/A
Standard Deviation	9.899495
Sample Variance	98
Kurtosis	#DIV/0!
Skewness	#DIV/0!

Range	1
Minimum	81
Maximum	82
Sum	163
Count	2
Confidence Level(95.0%)	6.353102
**UPPER CI**	**87.8531**
**LOWER CI**	**75.1469**

Range	11
Minimum	72
Maximum	83
Sum	155
Count	2
Confidence Level(95.0%)	69.88413
**UPPER CI**	**147.3841**
**LOWER CI**	**7.615874**

Range	14
Minimum	79
Maximum	93
Sum	172
Count	2
Confidence Level(95.0%)	88.94343
**UPPER CI**	**174.9434**
**LOWER CI**	**-2.94343**

ACCURACY (CNN)

Column1	
Mean	74
Standard Error	3.96297
Median	79
Mode	79
Standard Deviation	14.28869
Sample Variance	204.1667
Kurtosis	0.711166
Skewness	-0.90157
Range	53
Minimum	42
Maximum	95
Sum	962
Count	13
Confidence Level(95.0%)	8.634569

SENSITIVITY (CNN)

Column1	
Mean	69
Standard Error	5.161495
Median	72
Mode	69
Standard Deviation	18.61003
Sample Variance	346.3333
Kurtosis	3.405345
Skewness	-1.61469
Range	73
Minimum	20
Maximum	93
Sum	897
Count	13
Confidence Level(95.0%)	11.24593

SPECIFICITY (CNN)

Column1	
Mean	74
Standard Error	4.402796
Median	74
Mode	#N/A
Standard Deviation	15.87451
Sample Variance	252
Kurtosis	-0.7731
Skewness	-0.16957
Range	50
Minimum	49
Maximum	99
Sum	962
Count	13
Confidence Level(95.0%)	9.592869

**UPPER CI**	**82.63457**
**LOWER CI**	**65.36543**

ACCURACY (LDA)

Column1	
Mean	92
Standard Error	2
Median	92
Mode	#N/A
Standard Deviation	2.828427
Sample Variance	8
Kurtosis	#DIV/0!
Skewness	#DIV/0!
Range	4
Minimum	90
Maximum	94
Sum	184
Count	2
Confidence Level(95.0%)	25.41241
**UPPER CI**	**117.4124**
**LOWER CI**	**66.58759**

ACCURACY (RESPIRATORY TRACT)

Column1

**UPPER CI**	**80.24593**
**LOWER CI**	**57.75407**

SENSITIVITY (LDA)

Column1	
Mean	91.5
Standard Error	2.5
Median	91.5
Mode	#N/A
Standard Deviation	3.535534
Sample Variance	12.5
Kurtosis	#DIV/0!
Skewness	#DIV/0!
Range	5
Minimum	89
Maximum	94
Sum	183
Count	2
Confidence Level(95.0%)	31.76551
**UPPER CI**	**123.2655**
**LOWER CI**	**59.73449**

SENSITIVITY (RESPIRATORY TRACT)

Column1

**UPPER CI**	**83.59287**
**LOWER CI**	**64.40713**

SPECIFICITY (LDA)

Column1	
Mean	93
Standard Error	2
Median	93
Mode	#N/A
Standard Deviation	2.828427
Sample Variance	8
Kurtosis	#DIV/0!
Skewness	#DIV/0!
Range	4
Minimum	91
Maximum	95
Sum	186
Count	2
Confidence Level(95.0%)	25.41241
**UPPER CI**	**118.4124**
**LOWER CI**	**67.58759**

SPECIFICITY (RESPIRATORY TRACT)

Column1

Mean	84.33333	Mean	62.33333	Mean	74
Standard Error	5.333333	Standard Error	6.666667	Standard Error	2.081666
Median	79	Median	69	Median	73
Mode	79	Mode	69	Mode	#N/A
Standard Deviation	9.237604	Standard Deviation	11.54701	Standard Deviation	3.605551
Sample Variance	85.33333	Sample Variance	133.3333	Sample Variance	13
Kurtosis	#DIV/0!	Kurtosis	#DIV/0!	Kurtosis	#DIV/0!
Skewness	1.732051	Skewness	-1.73205	Skewness	1.15207
Range	16	Range	20	Range	7
Minimum	79	Minimum	49	Minimum	71
Maximum	95	Maximum	69	Maximum	78
Sum	253	Sum	187	Sum	222
Count	3	Count	3	Count	3
Confidence Level(95.0%)	22.94748	Confidence Level(95.0%)	28.68435	Confidence Level(95.0%)	8.956686
**UPPER CI**	**107.2808**	**UPPER CI**	**91.01768**	**UPPER CI**	**82.95669**
**LOWER CI**	**61.38585**	**LOWER CI**	**33.64898**	**LOWER CI**	**65.04331**

ACCURACY (ORAL CAVITIES)		SENSITIVITY (ORAL CAVITIES)		SPECIFICITY (ORAL CAVITIES)	
Column1		*Column1*		*Column1*	
Mean	74.4	Mean	67.4	Mean	72.8
Standard Error	5.537147	Standard Error	7.018547	Standard Error	9.046546
Median	76	Median	71	Median	82
Mode	#N/A	Mode	#N/A	Mode	#N/A
Standard Deviation	12.38144	Standard Deviation	15.69395	Standard Deviation	20.22869

Sample Variance	153.3
Kurtosis	-2.05883
Skewness	0.077236
Range	29
Minimum	61
Maximum	90
Sum	372
Count	5
Confidence Level(95.0%)	15.37359
**UPPER CI**	**89.77359**
**LOWER CI**	**59.02641**

Sample Variance	246.3
Kurtosis	-0.34222
Skewness	0.193537
Range	41
Minimum	48
Maximum	89
Sum	337
Count	5
Confidence Level(95.0%)	19.48661
**UPPER CI**	**86.88661**
**LOWER CI**	**47.91339**

Sample Variance	409.2
Kurtosis	-3.0707
Skewness	-0.51267
Range	42
Minimum	49
Maximum	91
Sum	364
Count	5
Confidence Level(95.0%)	25.11724
**UPPER CI**	**97.91724**
**LOWER CI**	**47.68276**

ACCURACY (SINUS & THYROID)

Column1	
Mean	76.66667
Standard Error	10.41367
Median	78
Mode	#N/A
Standard Deviation	18.037
Sample Variance	325.3333
Kurtosis	#DIV/0!
Skewness	-0.33083
Range	36
Minimum	58
Maximum	94

SENSITIVITY (SINUS & THYROID)

Column1	
Mean	89
Standard Error	4.50925
Median	93
Mode	#N/A
Standard Deviation	7.81025
Sample Variance	61
Kurtosis	#DIV/0!
Skewness	-1.70016
Range	14
Minimum	80
Maximum	94

SPECIFICITY (SINUS & THYROID)

Column1	
Mean	73.66667
Standard Error	12.41415
Median	74
Mode	#N/A
Standard Deviation	21.50194
Sample Variance	462.3333
Kurtosis	#DIV/0!
Skewness	-0.06974
Range	43
Minimum	52
Maximum	95

Sum	230	Sum	267	Sum	221
Count	3	Count	3	Count	3
Confidence Level(95.0%)	44.80639	Confidence Level(95.0%)	19.40174	Confidence Level(95.0%)	53.41377
**UPPER CI**	**121.4731**	**UPPER CI**	**108.4017**	**UPPER CI**	**127.0804**
**LOWER CI**	**31.86028**	**LOWER CI**	**69.59826**	**LOWER CI**	**20.25289**

Table S1. Computations for forest plot

S6. SUMMARY OF COMPUTATIONS OF DEEK'S FUNNEL PLOT

The computations that were performed for each Deeks' funnel plot are displayed in this section. It includes the total amount of observations required for the funnel plot and the regression statistics.

YEAR OF PUBLICATION

ACCURACY
SUMMARY OUTPUT

Regression Statistics	
Multiple R	0.240192
R Square	0.057692
Adjusted R Square	-0.88462
Standard Error	0.049497
Observations	3

SENSITIVITY
SUMMARY OUTPUT

Regression Statistics	
Multiple R	0.376404
R Square	0.14168
Adjusted R Square	-0.71664
Standard Error	0.190919
Observations	3

SPECIFICITY
SUMMARY OUTPUT

Regression Statistics	
Multiple R	0.909935
R Square	0.827982
Adjusted R Square	0.655963
Standard Error	0.035355
Observations	3

CANCER TYPES

ACCURACY

SUMMARY OUTPUT

Regression Statistics	
Multiple R	0.201528
R Square	0.040613
Adjusted R Square	-0.09644
Standard Error	0.171455
Observations	9

SENSITIVITY

SUMMARY OUTPUT

Regression Statistics	
Multiple R	0.039859
R Square	0.001589
Adjusted R Square	-0.14104
Standard Error	0.236877
Observations	9

SPECIFICITY

SUMMARY OUTPUT

Regression Statistics	
Multiple R	0.397317
R Square	0.157861
Adjusted R Square	0.037555
Standard Error	0.152772
Observations	9

CAD METHODS

ACCURACY

SUMMARY OUTPUT

Regression Statistics	
Multiple R	0.142857
R Square	0.020408
Adjusted R Square	-0.95918
Standard Error	0.113137
Observations	3

SENSITIVITY

SUMMARY OUTPUT

Regression Statistics	
Multiple R	0.197437
R Square	0.038981
Adjusted R Square	-0.92204
Standard Error	0.304056
Observations	3

SPECIFICITY

SUMMARY OUTPUT

Regression Statistics	
Multiple R	0.821995
R Square	0.675676
Adjusted R Square	0.351351
Standard Error	0.028284
Observations	3

VIVO

ACCURACY

SENSITIVITY

SPECIFICITY

SUMMARY OUTPUT	
Regression Statistics	
Multiple R	1
R Square	1
Adjusted R Square	65535
Standard Error	0
Observations	2

SUMMARY OUTPUT	
Regression Statistics	
Multiple R	1
R Square	1
Adjusted R Square	65535
Standard Error	0
Observations	2

SUMMARY OUTPUT	
Regression Statistics	
Multiple R	1
R Square	1
Adjusted R Square	65535
Standard Error	0
Observations	2

BAND

ACCURACY

SUMMARY OUTPUT	
Regression Statistics	
Multiple R	1
R Square	1
Adjusted R Square	65535
Standard Error	0
Observations	2

SENSITIVITY

SUMMARY OUTPUT	
Regression Statistics	
Multiple R	1
R Square	1
Adjusted R Square	65535
Standard Error	0
Observations	2

SPECIFICTY

SUMMARY OUTPUT	
Regression Statistics	
Multiple R	1
R Square	1
Adjusted R Square	65535
Standard Error	0
Observations	2

Table S2. Summary of computations of deek's funnel plot

S7. Deek's funnel plot computation of p-value

This section illustrates the estimations made to determine the p value required for the Deeks' funnel plot.

ANOVA (CANCER TYPES)

	df	*SS*	*MS*	*F*	*Significance F*
Regression	1	0.008711	0.008711	0.296328	0.603094
Residual	7	0.205778	0.029397		
Total	8	0.214489			

	Coefficients	*Standard Error*	*t Stat*	*P-value*	*Lower 95%*	*Upper 95%*	*Lower 95.0%*	*Upper 95.0%*
Intercept	0.668889	0.127795	5.234076	0.001207	0.366702	0.971076	0.366702	0.971076
X Variable 1	0.046667	0.085728	0.54436	0.603094	-0.15605	0.24938	-0.15605	0.24938

ANOVA (PUBLISHING YEAR)

	df	*SS*	*MS*	*F*	*Significance F*
Regression	1	0.00015	0.00015	0.061224	0.845579042
Residual	1	0.00245	0.00245		
Total	2	0.0026			

	Coefficients	*Standard Error*	*t Stat*	*P-value*	*Lower 95%*	*Upper 95%*	*Lower 95.0%*	*Upper 95.0%*
Intercept	0.855	0.144309	5.924799	0.106447	-0.978615848	2.688616	-0.97862	2.688616
X Variable 1	-0.015	0.060622	-0.24744	0.845579	-0.785272726	0.755273	-0.78527	0.755273

ANOVA (CAD METHODS)

	df	*SS*	*MS*	*F*	*Significance F*
Regression	1	0.000267	0.000267	0.020833	0.908742103
Residual	1	0.0128	0.0128		

Total	2	0.013067

	Coefficients	*Standard Error*	*t Stat*	*P-value*	*Lower 95%*	*Upper 95%*	*Lower 95.0%*	*Upper 95.0%*
Intercept	0.845	0.103923	8.131016	0.077904	-0.47546753	2.165468	-0.47547	2.165468
X Variable 1	-0.005	0.034641	-0.14434	0.908742	-0.445155843	0.435156	-0.44516	0.435156

Table S3. SUMMARY OF COMPUTATIONS OF DEEK'S FUNNEL PLOT
